# Torsion of Hydrosalpinx with Concurrent Acute Cholecystitis: Case Report and Review of Literature

**DOI:** 10.1155/2016/5424092

**Published:** 2016-12-14

**Authors:** Preeti R. John, Amelia M. Pasley

**Affiliations:** ^1^Baltimore VA Medical Center, 10 North Greene Street, 5C-119, Baltimore, MD 21201, USA; ^2^Department of Surgery, University of Maryland Medical Center, Baltimore, MD, USA; ^3^University of Maryland Medical Center, Baltimore, MD, USA

## Abstract

*Introduction.* Isolated torsion of the Fallopian tube is an uncommon cause of acute lower abdominal pain and can occur in women of all age groups. Cholecystitis is a frequent cause of upper abdominal pain. We present an unusual case with the presence of these two distinct pathological entities occurring concurrently in the same patient, causing simultaneously occurring symptoms. To our knowledge, this is the first reported presentation of such a case.* Methods.* We describe a 34-year-old premenopausal woman who presented with right sided upper and lower abdominal pain and nausea. Abdominal ultrasound (US) revealed acute cholecystitis. Vaginal US was suggestive of right hydrosalpinx. Intravenous antibiotics were administered and consent was obtained for operative intervention. During laparoscopy, the right Fallopian tube with hydrosalpinx was noted to be twisted three times. The right ovary appeared normal. The gall bladder wall was thickened and inflamed. Laparoscopic right salpingectomy and cholecystectomy were performed.* Results.* Surgical pathology revealed hydrosalpinx with torsion and acute calculous cholecystitis. The patient had an uneventful postoperative course and was discharged home on the first postoperative day. Her symptoms resolved after the procedure.* Conclusions.* In women with abdominal pain, both gynecologic and nongynecologic etiologies should be considered in the differential diagnoses. Concurrent presence of symptomatic gynecologic and nongynecologic intra-abdominal pathology is rare. Isolated Fallopian tube torsion is rare and is associated most often with hydrosalpinx. Some torqued Fallopian tubes can be salvaged. Laparoscopy is useful in management of both Fallopian tube torsion and cholecystitis.

## 1. Introduction

Isolated torsion of the Fallopian tube (also called “salpinx”) without an ovarian abnormality is an uncommon cause of acute lower abdominal pain. The incidence is estimated to be 1 in 500,000 women [1]. It is most often found in women of reproductive age but has also been reported in prepubertal and postmenopausal women. “Hydrosalpinx,” a blocked, fluid-filled Fallopian tube, is the most common lesion associated with fallopian tube torsion [2].

Acute cholecystitis or inflammation of the gallbladder occurs most commonly (96% of the time) due to cholelithiasis (gallstones) [3]. Cholelithiasis is common, affecting 10–15% of the adult population [4]. 1.8 million ambulatory care visits each year are estimated to be a result of gallstone disease, making it the leading cause of hospital admissions related to gastrointestinal problems [5]. The majority (75%) of patients with acute cholecystitis present with right upper quadrant pain, nausea, emesis, or dyspepsia. Diagnosis is most commonly made with abdominal ultrasound. The treatment of choice is laparoscopic cholecystectomy [6].

This case report describes the unique clinical presentation and surgical management of a patient with two distinct gynecologic and nongynecologic pathologic entities occurring concurrently and causing symptoms: torsion of hydrosalpinx and acute calculous cholecystitis.

## 2. Case Report

A 34-year-old nulliparous female presented to our institution with right sided upper and lower abdominal pain. She stated that it woke her up from sleep and had been persistent for 12 hours. She described the pain as colicky and radiating from her right upper abdomen to her groin and thigh. She denied any association with food, fever, chills, nausea, and alteration in micturition or bowel habits. She had regular menstrual cycles and no history of sexually transmitted diseases.

She had a history of intermittent abdominal pain and had been diagnosed previously with chronic constipation and cholelithiasis. She denied any previous surgery. Her home medications consisted of oral contraceptive pills and polyethylene glycol.

On physical examination she was tender to palpation in the right upper and lower quadrants of her abdomen, with a positive Murphy's sign. Her initial WBC count was 12.5 × 10^9^/L. Urine analysis and pregnancy test were negative. Abdominal ultrasound showed acute cholecystitis and pelvic ultrasound findings suggested a right sided hydrosalpinx. A CT scan was also obtained and confirmed ultrasound results. Intravenous antibiotics were administered and the patient was taken to the operating room. The following were visualized during laparoscopy: right hydrosalpinx with torsion (3 twists of Fallopian tube observed) and a normal right ovary. The gallbladder appeared inflamed. A laparoscopic right salpingectomy and cholecystectomy were performed, respectively, by the Gynecology and General Surgery teams working together in the operating room. Consent for both procedures had been obtained preoperatively.

Pathology revealed torsion of the right Fallopian tube: a twisted right hydrosalpinx with hemorrhage within, vascular congestion, small benign mesothelial inclusion cysts, and acute, calculous cholecystitis.

The patient had an uneventful postoperative course and was discharged on the first postoperative day. Symptoms resolved after the procedure.

## 3. Discussion

Common causes of lower abdominal pain include appendicitis, pyelonephritis, cystitis, biliary or renal colic, perforated or obstructed intestine, mesenteric lymphadenitis, strangulated hernia, inflammatory bowel disease, or diverticulitis. Gynecologic causes should also be considered and include torsion or rupture of adnexal structures (e.g., twisted ovarian cyst, ruptured ovarian follicle, ruptured corpus luteum cyst), pelvic inflammatory disease, or ectopic pregnancy. Isolated torsion of the fallopian tube without an ovarian abnormality is an uncommon cause of acute lower abdominal pain and requires a high index of suspicion to be recognized and treated.

The Fallopian tube is also called “salpinx.” Causative mechanisms underlying Fallopian tube torsion are not fully understood. Various predisposing factors and theories have been proposed (Table 1) [7, 8]. Torsion can also affect previously healthy tubes [9]. It has been proposed that the incidence of Fallopian tube torsion is more common on the right side: it is possible that the sigmoid colon may prevent torsion on the left, and surgeons are more likely to operate if women complain of right sided abdominal pain to exclude appendicitis [10].

Hydrosalpinx (blocked, fluid-filled Fallopian tube) is the most common abnormality associated with Fallopian tube torsion [2]. Hydrosalpinx may be unilateral or bilateral. Etiology of hydrosalpinx includes pelvic inflammatory disease (PID) causing tubal occlusion, previous abdominal surgery, endometriosis, ectopic pregnancy, and congenital malformation. A hydrosalpinx usually is asymptomatic; however, it may be associated with chronic pelvic pain, dyspareunia, and a sense of pelvic pressure [11].

Ultrasound is the standard imaging modality in the evaluation of an adnexal mass and has been shown to be helpful in the diagnosis of a twisted Fallopian tube. An ultrasound image of hydrosalpinx may appear as an elongated, convoluted cystic mass, tapering as it nears the uterine cornua, with the ipsilateral ovary separate from the mass. Doppler evaluation could be helpful with this diagnosis if high impedance or absence of flow in a tubular structure is noted. The characteristic “whirlpool sign” has been suggested to be specific for Fallopian tube torsion, as well as evidence of reversal or absent flow in color Doppler of the affected tube, due to obstruction of blood supply [12].

Isolated tubal torsion can be managed with either de-torsion or simple salpingectomy. The operative approach should ideally be laparoscopic because of smaller incisions, less blood loss, less pain, and shorter hospital stay associated with laparoscopic procedures. Laparoscopy serves not only as a diagnostic tool but also as an excellent therapeutic strategy [13]. Even during pregnancy, laparoscopic approach is feasible during first and second trimesters [14].

Several authors advocate de-torsion of the pedicle with preservation of adnexal structures [15, 16]. Timely diagnosis may allow conservative management of torsion in select cases, with untwisting and conservation of tube and preservation of fertility [10]. Adnexal de-torsion should be performed as early as possible to avoid irreversible damage to the tissue.

In premenarcheal and reproductive age women, preservation of future conception capacity with restorative surgery should be considered. A two-staged oviduct-sparing procedure has been described for Fallopian tube torsion: laparoscopic de-torsion and puncture of the hydrosalpinx for cytologic and bacteriologic analysis (to rule out malignancy and infectious disease), with evacuation of the affected Fallopian tube. Second-look laparoscopic and salpingoscopic evaluation should be performed several weeks after the first procedure. If the tube is judged to be viable, tubal salvage with “neosalpingostomy” is performed and consists of surgical repair of the Fallopian tube to allow future pregnancy [17, 18]. The incidence of recurrent torsion following conservative management is unknown. Therapeutic options for tubal torsion should be considered on a case-by-case basis, as not all torqued Fallopian tubes can be salvaged.

Frequent causes of upper abdominal pain include peptic ulcer disease, gastroesophageal reflux disease (GERD), biliary colic, cholecystitis, and pancreatitis.

Cholecystitis may develop in association with gallstones (“calculous” cholecystitis), or without (“acalculous” cholecystitis). Gallstones are prevalent in 10–15% of the adult population [19]. More than 80% of patients with gallstones are asymptomatic [20]. Acute cholecystitis develops in 1–3% of patients with gallstones. Classic acute cholecystitis is characterized by right upper quadrant pain, fever, and leukocytosis, although only 8% of patients have this triad. Tokyo guidelines have developed diagnostic and severity assessment criteria [21]. The diagnostic criteria for acute cholecystitis are one local sign of inflammation (Murphy sign: mass, pain, and/or tenderness in right upper quadrant), one systemic sign of inflammation (fever, elevated C-reactive protein level, and elevated white blood cell count), and confirmatory imaging findings.

Ultrasound is the most frequently performed imaging modality for right upper quadrant pain and yields a sensitivity of 88% and a specificity of 80% in the diagnosis of acute cholecystitis [22]. It should be considered the primary imaging technique for patients clinically suspected of having acute cholecystitis [23].

Management of acute cholecystitis commonly involves antibiotics and laparoscopic cholecystectomy, but percutaneous cholecystostomy may be considered in high risk patients who are unlikely to tolerate general anesthesia [24, 25].

## 4. Conclusion

This case is unusual because of the concurrent presence of two distinct gynecologic and nongynecologic pathological entities, torsion of hydrosalpinx and acute cholecystitis, causing symptoms simultaneously. In women with lower abdominal pain, gynecological causes should always be included in the differential diagnoses. Hydrosalpinx is a common cause of Fallopian tube torsion. Some torqued Fallopian tubes can be salvaged with de-torsion and neosalpingostomy, but this should be attempted only in selected cases. Laparoscopy is useful in diagnosis and treatment of both torsion of hydrosalpinx and acute cholecystitis.

## Figures and Tables

**Table 1:** Fallopian tube torsion: predisposing factors and theories for etiology [7, 8].

Intrinsic (directly related to Fallopian tube)	Inflammatory disease, hydrosalpinx, hematosalpinx
	Tubal neoplasms, for example, cyst of Morgagni, adenofibroma
	Congenital abnormalities, for example, stricture
	Venous congestion leading to spiraling of veins
	Tubal surgery: tubal ligation, reconstruction
	Physiological abnormalities: hypermobility, spasm, excessive length, or tortuosity
	Abnormal peristalsis due to autonomic dysfunction, drugs
	Anatomic malformations in tube or mesosalpinx
Extrinsic	Ovarian or paraovarian masses or cysts
	Uterine enlargement (pregnancy, tumors)
	Adhesions
	Mechanical factors/trauma to pelvic organs
