# Erratum to “Torsion of Hydrosalpinx with Concurrent Acute Cholecystitis: Case Report and Review of Literature”

**DOI:** 10.1155/2017/7865624

**Published:** 2017-12-28

**Authors:** Preeti R. John, Amelia M. Pasley

**Affiliations:** ^1^Baltimore VA Medical Center, 10 North Greene Street, 5C-119, Baltimore, MD 21201, USA; ^2^Department of Surgery, University of Maryland Medical Center, Baltimore, MD, USA; ^3^University of Maryland Medical Center, Baltimore, MD, USA

In the article titled “Torsion of Hydrosalpinx with Concurrent Acute Cholecystitis: Case Report and Review of Literature” [1], the figures of the study were missing. The figures are as follows.

## Figures and Tables

**Figure 1:**
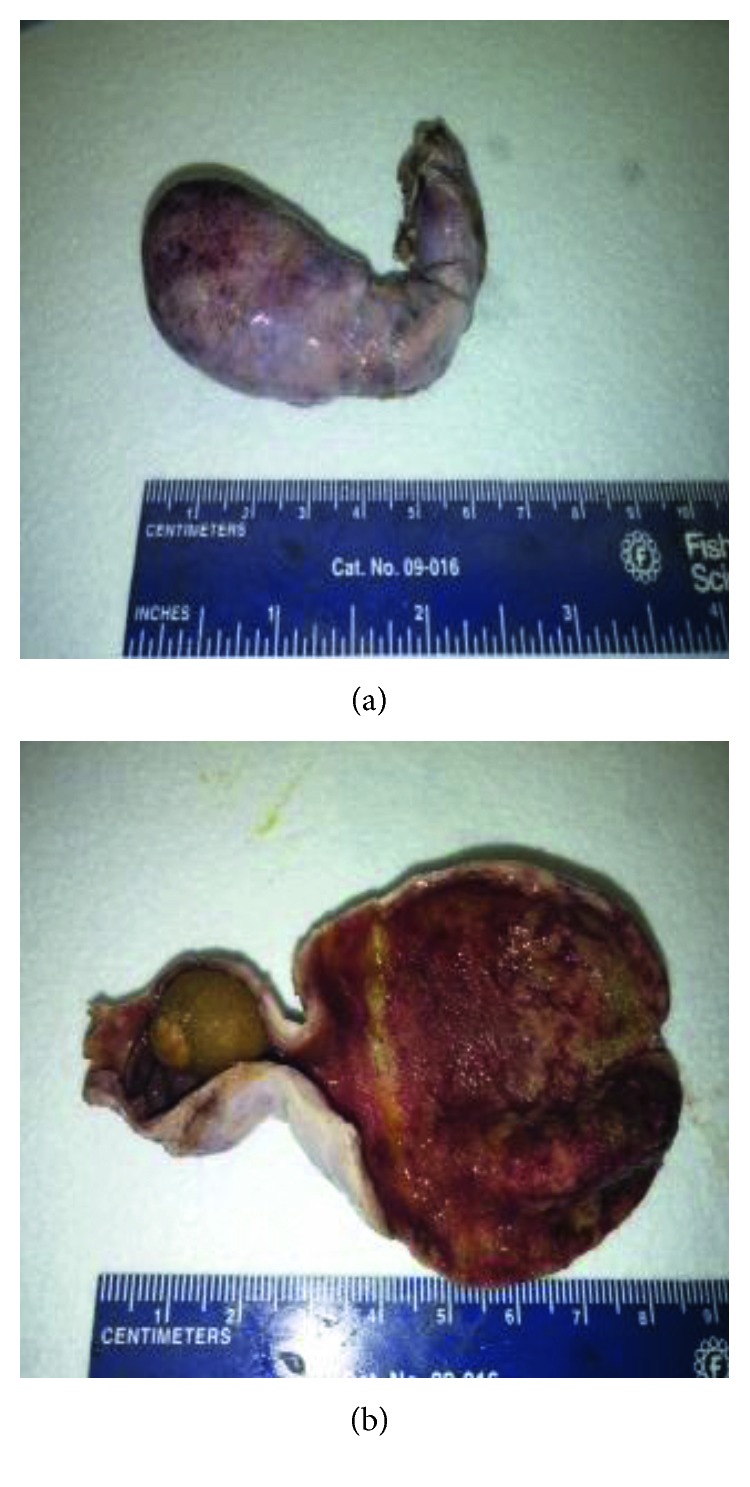
(a) Gall bladder. (b) Open gall bladder with stone impacted at infundibulum.

**Figure 2:**
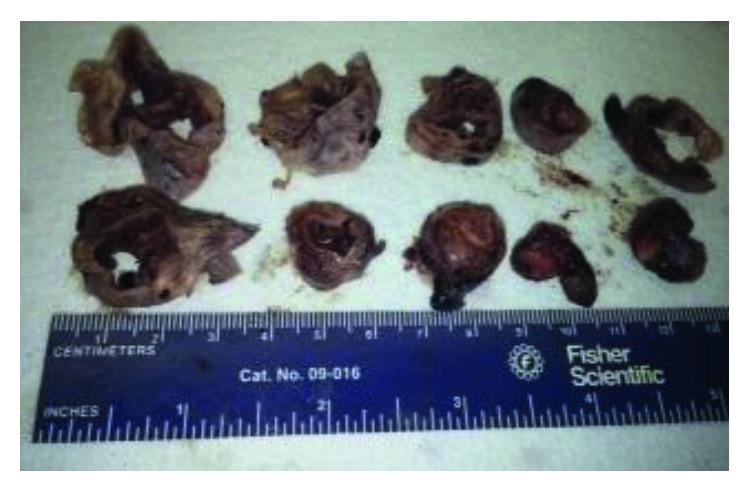
Fallopian tube with hydrosalpinx.
